# Umbilical Cord Blood Platelet Lysate Eyedrops for the Treatment of Severe Ocular Surface Disorders in Graft vs. Host Disease Patients: Clinical Study

**DOI:** 10.3390/life14101268

**Published:** 2024-10-05

**Authors:** Caterina Gagliano, Roberta Foti, Marco Zeppieri, Antonino Maniaci, Salvatore Lavalle, Giuseppa Tancredi, Giuseppe Gagliano, Alessandro Avitabile, Ludovica Cannizzaro, Rosario Foti

**Affiliations:** 1 Department of Medicine and Surgery, University of Enna “Kore”, Piazza dell’Università, 94100 Enna, Italy; caterina.gagliano@unikore.it (C.G.);; 2 Division of Rheumatology, A.O.U. “Policlinico-San Marco”, 95123 Catania, Italyrosfoti5@gmail.com (R.F.); 3 Department of Ophthalmology, University Hospital of Udine, 33100 Udine, Italy; 4 Sciacca Cord Blood Bank, 92019 Sciacca, Italy; 5 Faculty of Medicine, University of Catania, 95123 Catania, Italy

**Keywords:** graft-versus-host disease, autoimmunity, umbilical cord blood serum, ocular surface disorders, corneal ulcers

## Abstract

*Background:* Graft-versus-host disease (GvHD) is an overactive systemic inflammatory response that can arise following allogeneic hematopoietic stem cell transplantation (HSCT). This condition occurs when the transplanted donor immune cells recognize the recipient’s tissues as foreign and trigger an immune response against them. The ocular surface (eyelids, conjunctiva, meibomian glands, lacrimal glands, and cornea) is particularly involved in GvHD, and its response to existing treatments, including potent immunosuppressants and new targeted therapies, is undesirable, with such treatments often being ineffective. Human allogeneic umbilical cord blood platelet lysate stands out as a potent adjunct to conventional therapies for ocular surface disorders related to severe Dry Eye Disease. This study aimed to evaluate the safety and efficacy of umbilical cord blood platelet lysate eyedrops for the treatment of severe ocular surface disorders in graft-versus-host disease patients who have received previous unsuccessful treatments. *Methods:* This study was a prospective, non-comparative, interventional case series study involving 22 patients (10 females and 12 males) aged 25–46 years with severe ocular surface disorders that were unresponsive to standard treatments. The GvHD patients were categorized based on the severity of their ocular surface disorders into three groups: Group I: five patients with severe Dry Eye Disease and filamentary keratitis; Group II: eight patients suffering from severe blepharo-kerato-epitheliopathy; Group III: nine patients with corneal ulcers. Fresh umbilical cord blood (UCB) was obtained from healthy donors and subjected to centrifugation using a novel PRP preparation kit provided by Sciacca (AG) Cord blood bank, Italy in a one-step process. In all groups, the outcomes before and after treatment were evaluated by means of the OSDI (Ocular Surface Disease Index), SANDE (Symptom Assessment in Dry Eye) questionnaire, VAS (Visual Analogue Scale), slit lamp examination, Esthesiometry, Lissamine Green Staining, the NIBUT (Non-Invasive Break-Up Time) and BUT, fluorescein staining with digital photography and Oxford classification, the Schirmer Test, the Best Corrected Visual Acuity (BCVA), and Meibography. In Group III at each evaluation time, the size of the ulcer and its relative reduction compared to the baseline size were recorded. Clinical variables, such as corneal inflammation, conjunctivalization, corneal neovascularization, or pain, were also considered individually. *Results:* We observed a significant improvement in the SANDE, VAS, and OSDI scores; Schirmer Test; BUT; BCVA; and Oxford classification after treatment with allogeneic cord blood serum eyedrops. Nevertheless, pain and inflammation reduced markedly over time until complete healing in all cases. The mean reduction in the ulcer surface area (compared to baseline values) was significantly higher at all assessment points (*p* = 0.001 for day 7 and *p* < 0.001 for subsequent time points every 30 days for 90 days). At the last check-up (after 90 days of treatment), the number of ulcers (Group III, nine patients) with a reduction in size of greater than 50% was eight (88.8%), of which seven ulcers were completely healed. None of the patients experienced treatment-related local or systemic adverse events. In this study, using a relatively large number of cases, we demonstrated that the use of umbilical cord blood platelet lysate eyedrops is a safe, feasible, and effective curative approach for severe ocular surface disease in patients with GvHD. *Conclusions:* Our pilot study highlights the remarkable effectiveness of allogeneic cord blood serum eyedrops in patients with severe ocular surface disorders following GvHD who have shown an inadequate response to the usual treatments. It is mandatory to design future studies on the efficacy of this therapeutic approach for acute ocular, mucosal, and cutaneous GvHD.

## 1. Introduction

Graft-versus-host disease (GvHD) is an overactive systemic inflammatory response that can develop following allogeneic hematopoietic stem cell transplantation (HSCT) [1]. This condition occurs when the transplanted donor immune cells recognize the recipient’s tissues as foreign and mount an immune response against them [2]. This immune-mediated attack results in tissue damage and inflammation, adding to the complexity and severity of the disease [3]. GvHD is a significant complication of HSCT, affecting approximately 40% to 60% of patients undergoing this procedure [4]. The condition manifests in a spectrum of severity, ranging from mild symptoms to life-threatening disease, with a mortality rate that can reach up to 15%, depending on the severity and organs involved [5]. GvHD is a multifaceted disease that presents in both acute and chronic forms [6].

Indeed, acute graft-versus-host disease (a GvHD) is a serious complication that can occur after an allogeneic HSCT [7]. This condition happens when the donor’s immune cells, particularly T cells, recognize the recipient’s tissues as foreign and attack them [2]. The skin, liver, and gastrointestinal tract are the most commonly affected organs, though other tissues can also be involved [8]. A GvHD typically appears within the first 100 days post-transplant, which helps distinguish it from chronic GvHD that occurs later [9].

The onset of a GvHD begins with tissue damage caused by the conditioning regimen given before the transplant, which often includes chemotherapy and/or radiation. This damage leads to the release of inflammatory cytokines and exposes host antigens that donor T cells see as foreign. The activation of these T cells triggers a series of immune reactions, including the release of more cytokines like IL-2, TNF-alpha, and IFN-gamma, which further drive the immune response and tissue damage [10].

Several factors can increase the risk of developing a GvHD, including the degree of mismatch in human leukocyte antigens (HLAs) between the donor and recipient, the use of peripheral blood stem cells (which contain more T cells compared to bone marrow or cord blood), and the intensity of the conditioning regimen. Additionally, genetic variations in both the donor and recipient can influence the risk and severity of a GvHD [11].

The clinical presentation of a GvHD depends on the organs involved. The skin is most commonly affected and often exhibits the first sign, presenting as a maculopapular rash that can escalate to severe erythroderma or blistering. Gastrointestinal symptoms may include nausea, vomiting, abdominal pain, diarrhea, and, in severe cases, gastrointestinal bleeding. Liver involvement typically results in jaundice, elevated bilirubin levels, and an enlarged liver. The severity of a GvHD is graded based on the extent of organ involvement, with grades I to IV indicating increasing severity. Higher grades generally suggest a poorer prognosis [12].

The mainstay of a GvHD treatment is immunosuppression, which aims to control the donor T-cell response while preserving the graft-versus-leukemia (GVL) effect, crucial for preventing cancer relapse in patients with blood cancers. The first-line treatment is typically corticosteroids like methylprednisolone, which reduce inflammation and suppress T-cell activity. However, only about half of a GvHD patients fully respond to steroids, making additional treatments necessary for those with steroid-refractory disease [13].

For patients who do not respond to corticosteroids, second-line therapies are available. Ruxolitinib, a JAK1/2 inhibitor, has been effective in treating steroid-refractory a GvHD by targeting cytokine signaling pathways involved in inflammation [11]. Mesenchymal stem cells (MSCs), which have immunomodulatory properties, have also shown some success, particularly in patients with gastrointestinal involvement [10]. Extracorporeal photopheresis (ECP), which induces apoptosis in alloreactive T cells, is another option, especially when other treatments have failed [14]. Additionally, other immunosuppressants like tacrolimus, sirolimus, and mycophenolate mofetil are often used with corticosteroids to enhance treatment outcomes [15].

New therapies are focusing on more targeted approaches for managing a GvHD. Blocking specific cytokines such as IL-6 and TNF-alpha with monoclonal antibodies like tocilizumab and infliximab has shown promise in early clinical trials [16]. The use of regulatory T cells (Tregs), which can suppress the alloreactive T cells responsible for a GvHD while maintaining the GVL effect, is also being explored [17]. Additionally, the role of gut microbiota in immune modulation has led to the investigation of fecal microbiota transplantation (FMT) as a potential treatment for gastrointestinal a GvHD [18].

The outlook for a GvHD varies widely depending on the severity of the disease and how well the patient responds to treatment. While mild cases may resolve with minimal long-term effects, severe or refractory cases are associated with significant illness and risk of death. Research continues with the aim to better understand the mechanisms of a GvHD and to develop more effective and less harmful treatments. The integration of novel biomarkers into clinical practice could improve risk assessment and enable earlier, more targeted interventions, ultimately improving outcomes for patients undergoing HSCT [19].

Chronic GvHD, on the other hand, may develop later and can involve a broader range of organs, leading to long-term complications and significant morbidity [9]. The disease can affect various organs, including the lungs [20], hepatobiliary system [21], musculoskeletal system [22], gastrointestinal tract [23], skin [24], and eyes [25], each presenting unique clinical challenges that require tailored therapeutic strategies [26].

Chronic GvHD is a common and serious complication that occurs after allogeneic HSCT, a procedure often used to treat blood cancers and other serious conditions [4]. Despite improvements in HSCT, it remains a leading cause of long-term illness and death among survivors [27]. This condition can affect many organs and requires long-term treatment with immunosuppressive drugs, which can have severe side effects and reduce quality of life [28].

Chronic GvHD is complex, involving several overlapping biological processes such as inflammation, thymus damage, and tissue repair that can lead to fibrosis [29]. The risk of developing chronic GvHD is influenced by factors like the characteristics of the donor and recipient, the type of transplant, and the treatment used before the transplant. The use of peripheral blood stem cells or donors who are not fully matched or are unrelated increases the risk [30].

The main treatment is corticosteroids, despite their significant side effects [31]. However, advances in understanding the disease have led to the development of new treatments that target specific disease processes [32]. Drugs like ibrutinib, ruxolitinib, and belumosudil are being tested in clinical trials and show promise as more targeted and less toxic options [33].

Efforts are also being made to improve how this condition is diagnosed and assessed, with standardized guidelines helping to make clinical evaluations more consistent [34]. Research is ongoing to find biomarkers and other early signs of chronic GvHD, aiming to create personalized treatments that better fit each patient’s situation [35].

Overall, while chronic GvHD is still difficult to manage, research is leading to better and safer treatment options, with a focus on better understanding the disease and improving the long-term health and quality of life of patients [36]. Acute and chronic GvHD are driven by distinct processes. The onset of acute GvHD is linked to donor T cells reacting against the recipient’s antigens. In cases with an MHC mismatch, donor CD4+ T cells can trigger this condition [7]. For most current cases involving MHC-matched transplants, donor T-cell activation involves three steps: recognition of antigens, co-stimulatory signals, and cytokine-driven differentiation and expansion [37]. Initially, antigen-presenting cells (APCs) are stimulated by factors such as underlying illness, past infections, or tissue damage from the conditioning regimen, leading to the release of DAMPs and PAMPs [38]. Following this, donor T cells are activated, multiply, and differentiate, with co-stimulatory signals between CD28 on T cells and CD80 or CD86 on APCs. This activation results in the production of cytokines like IL-2 and IFN-γ, which are crucial for the development of acute GvHD. The final stage involves T cells causing tissue injury and cell death [39]. On the other hand, the mechanisms behind chronic GvHD are less clear. Various immune cells, including regulatory T cells (Tregs), B cells, and effector T cells, are thought to play a role. Patients with chronic GvHD often have fewer Tregs compared to controls, while an increase in B cells has been observed in this condition.

Moreover, the management of GvHD is inherently complex due to the diverse clinical manifestations and the involvement of multiple organ systems. A multidisciplinary approach is essential to effectively treat patients, involving specialists in hematology, oncology, gastroenterology, dermatology, ophthalmology, and other relevant fields [40]. Despite the use of potent immunosuppressants and the development of new targeted therapies, controlling the disease remains challenging, particularly when it affects the ocular surface [41].

The ocular surface, which includes the eyelids, conjunctiva, meibomian glands, lacrimal glands, and cornea, is particularly vulnerable in GvHD [42]. The predominant manifestation of ocular GvHD is Dry Eye Disease (DED), primarily driven by fibrosis in the lacrimal glands [43]. This fibrosis, consistent with chronic GvHD, leads to reduced tear production and increased evaporation due to tear film disruption. Immunohistochemical studies revealed activated CD34+ fibroblasts and periductal CD8+ T-cell infiltration in ocular GvHD lacrimal glands, with fibrosis correlating with symptom severity [44]. It is hypothesized that donor-derived CD4+ T cells activate fibroblasts, perpetuating inflammation and fibrosis [45].

Meibomian gland dysfunction (MGD) is another ocular GvHD-associated DED manifestation, occurring in 47.5% to 80% of patients, even pre-transplant [46]. MGD arises from structural and functional gland changes, with clinical signs including orifice obstruction, vascular abnormalities, and acinar cell atrophy [47]. Ocular GvHD patients often exhibit more severe MGD than do those with Sjögren’s syndrome or non-ocular GvHD MGD [48]. A pre-transplantation loss of meibomian gland area may predict ocular GvHD, though this is controversial [49].

Corneal involvement in ocular GvHD is common, with superficial punctate keratopathy and filamentary keratitis frequently observed [50]. Severe cases may develop persistent epithelial defects (PEDs) or corneal perforation [51]. Ocular GvHD-related conjunctival fibrosis can cause cicatricial changes, exacerbating DED [52]. Molecular studies indicate that conjunctival fibrosis involves epithelial-to-mesenchymal transition (EMT), with shared mechanisms in lacrimal glands [53].

Squamous cell metaplasia and decreased goblet cell density in the conjunctiva further compromise ocular surface lubrication and protection [54]. Other ocular complications include ocular hypertension, glaucoma, episcleritis, scleritis, uveitis, and retinal conditions [55]. The risk factors for ocular GvHD development are varied, with preventative strategies focusing on proper HLA matching and T-cell activity modulation using agents like calcineurin inhibitors, methotrexate, and targeted therapies [56].

In recent years, there has been increasing interest in using human umbilical cord blood platelet lysate as a supplement to conventional therapies for treating ocular surface disorders, particularly in patients with severe DED associated with GvHD [57]. Evidence suggests that this therapy can accelerate ocular surface regeneration, improve epithelial integrity, and enhance both patient experiences and clinical outcomes [58]. Studies have shown that the lysate’s anti-inflammatory properties are particularly effective in treating corneal ulcers, helping to reduce inflammation and promote epithelial healing, which contributes to better ocular surface stability [59].

Overall, umbilical cord blood platelet lysate shows promise in managing ocular surface disorders in GvHD patients, but further research and larger clinical trials are needed to confirm its efficacy and safety.

This study aimed to evaluate the safety and efficacy of umbilical cord blood platelet lysate eyedrops in the treatment of severe ocular surface disorders in GvHD patients who have previously experienced unsuccessful treatments. The potential of this therapy to improve clinical outcomes in these challenging cases represents a significant advancement in the management of ocular GvHD, offering hope for improved patient care and quality of life.

## 2. Materials and Methods

### 2.1. Study Design

This study was designed as a prospective, non-comparative, interventional case series conducted at San Marco Hospital in Catania, Italy. Informed consent was obtained from all patients.

### 2.2. Participants, Setting, and Inclusion Criteria

The study included 22 patients (10 females, 12 males) aged between 25 and 46 years who presented with severe ocular surface disorders unresponsive to standard treatment modalities.

The patients in this study were receiving standard systemic therapy for GvHD, which typically included a combination of corticosteroids, such as prednisone, and calcineurin inhibitors, like cyclosporine or tacrolimus, aimed at controlling immune-mediated damage. In cases of steroid-refractory GvHD, additional agents such as mycophenolate mofetil or rituximab were administered to further modulate the immune response.

- Inclusion criteria: severe Dry Eye Disease (DED), blepharo-kerato-epitheliopathy, or corneal ulcers that had not responded to prior treatments.
- Exclusion criteria: patients with active bacterial, viral, or fungal infections, as identified by conjunctival swab, and those who had undergone experimental treatments within the past 6 months.
- Patients were stratified into three groups based on their condition:
- Group I: 5 patients with severe Dry Eye Disease and filamentary keratitis;
- Group II: 8 patients with severe blepharo-kerato-epitheliopathy;
- Group III: 9 patients with corneal ulcers (Table 1).

### 2.3. Umbilical Cord Blood Collection and Processing

Fresh umbilical cord blood (UCB) was packaged and supplied by Sciacca (AG) Cord Blood Bank, Italy. Cord blood banks are public health facilities that collect, store, and distribute cord blood hematopoietic stem cells. These banks adhere to strict quality and safety standards for human use to ensure that all procedures meet regulatory and ethical guidelines [60]. These standards include ensuring that the environment for collecting, processing, and storing cord blood is sterile and that the procedures used minimize any risk of contamination [61].

Human umbilical cord blood serum (HUCBS) was retrieved after obstetric data collection (gestational age of the mother, sex and birth weight of the newborn, duration of labor, Apgar scores 9 or 10, and mode of delivery). All steps from recruitment to the processing and registration of the cord blood were performed according to guidelines provided by Italian regulations. This study used cord blood units not suitable for hematopoietic stem cell transplantation. The cord blood units were then sent to the processing facility laboratory at Sciacca (AG) Cord Blood Bank for further preparation procedures. The units underwent a series of checks and tests to establish the blood characteristics and their suitability for preservation as blood components for therapeutic use. The preparation of cord blood eye drops was performed under sterile conditions.

Collected from healthy donors and subsequently processed through centrifugation using an innovative platelet-rich plasma (PRP) preparation kit in a streamlined, single-step procedure, the resulting platelet lysate was then formulated into single-dose eye drops; these were administered to the patients, who were instructed to apply the drops six times daily.

### 2.4. Outcome Measures

The study’s outcomes were rigorously assessed both before and after the intervention using a variety of clinical and patient-reported measures. These included the following:

- The Ocular Surface Disease Index (OSDI);
- The Symptom Assessment in Dry Eye (SANDE) questionnaire;
- The Visual Analogue Scale (VAS) score;
- Slit lamp examination;
- Esthesiometry;
- Lissamine Green Staining;
- The Non-Invasive Break-Up Time (NIBUT) and Break-Up Time (BUT);
- Fluorescein staining with digital photography and Oxford classification;
- The Schirmer Test;
- The Best Corrected Visual Acuity (BCVA);
- Meibography.

In Group III, which consisted of patients with corneal ulcers, additional metrics were recorded at each evaluation point, specifically the size of the ulcer and the relative reduction in size compared to the baseline measurement. Overall, the patients received a total of 90 kits of platelet lysate eye drops. In addition to the primary outcomes, other clinical variables such as corneal inflammation, conjunctivalization, corneal neovascularization, and pain were also individually evaluated to assess the therapeutic impact of the treatment.

### 2.5. Statistical Analysis

Data were analyzed using SPSS software version 25.0 (IBM, Armonk, NY, USA). Descriptive statistics were used to summarize the data. Continuous variables are expressed as the mean ± standard deviation (SD). A paired *t*-test was used to compare pre- and post-treatment outcomes, and *p* < 0.05 was considered statistically significant.

## 3. Results

We observed a significant improvement in the SANDE, VAS, and OSDI scores, as well as in clinical parameters such as the Schirmer Test, BUT (Break-Up Time), BCVA (Best Corrected Visual Acuity), and Oxford classification following treatment with allogeneic cord blood serum eye drops. These improvements were consistent across all patient groups, regardless of the initial severity of their ocular surface disorders.

In Group I (patients with severe Dry Eye Disease), the OSDI scores decreased by an average of 45% after 30 days of treatment, indicating a marked subjective improvement in symptoms. Additionally, the Schirmer Test results showed an average 30% increase in tear production, which was particularly notable in Group III (patients with corneal ulcers), where significant reductions in ulcer size were also observed.

For Group II (patients with blepharo-kerato-epitheliopathy), we noted a 75% reduction in corneal neovascularization and conjunctivalization, confirmed by slit lamp examination and by digital photography after fluorescein staining. Furthermore, the BUT increased by an average of 3.5 s across all groups, reflecting a significant improvement in tear film stability.

**Figure 1:**
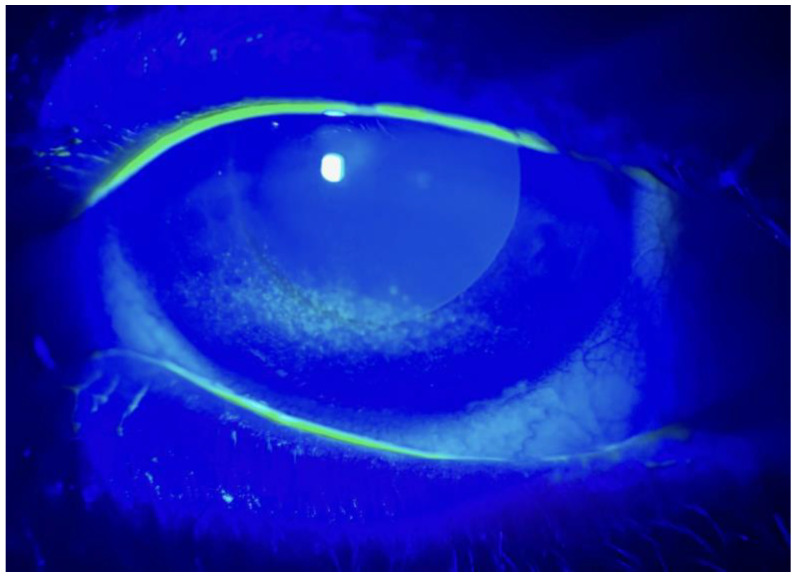
Meibomian gland dysfunction-related kerato-epitheliopathy: before treatment. We can observe deep damage to the epithelial layer, highlighted by intense fluorescein staining.

**Figure 2:**
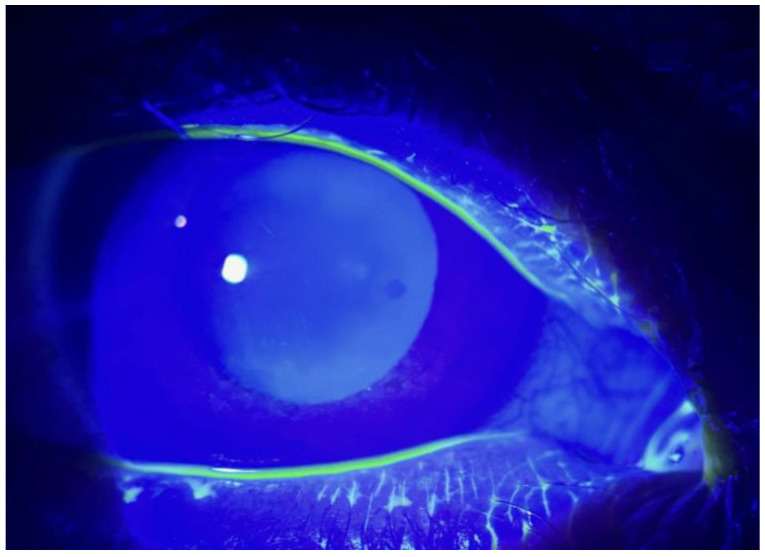
Meibomian gland dysfunction-related kerato-epitheliopathy after 7 days of treatment. We can observe an almost complete resolution of the epithelial damage and a notable attenuation of the fluorescein staining.

**Figure 3:**
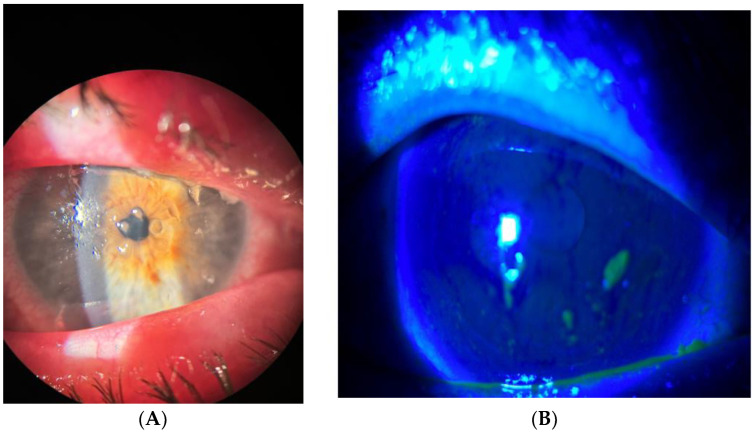
(**A**,**B**) Filamentary keratitis before treatment.

**Figure 4:**
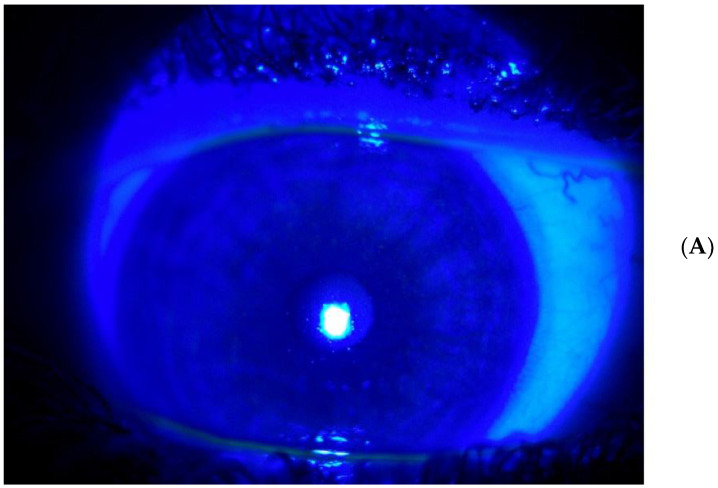

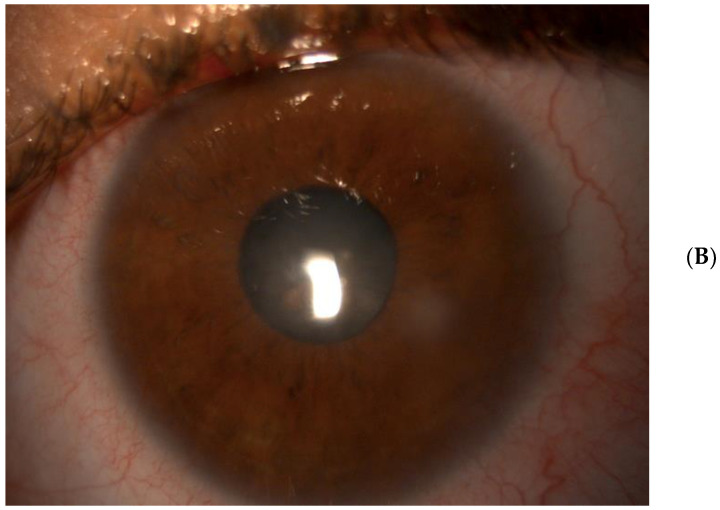
(**A**,**B**) Filamentary keratitis after one month of treatment.

In Group III, which included patients with corneal ulcers, the reduction in the ulcer surface area was significant at all time points (*p* = 0.001 at day 7), and by the final follow-up at 90 days, 88.8% of patients had more than a 50% reduction in ulcer size, with seven out of nine ulcers completely healed. Additionally, improvements in the BCVA were observed in six patients in Group II and four patients in Group III, with an average gain of 1.2 lines on the decimal scale (Figure 1, Figure 2, Figure 3 and Figure 4).

Clinical parameters such as corneal inflammation, conjunctivalization, corneal neovascularization, and pain were assessed individually. Notably, both pain and inflammation decreased significantly over time, with complete resolution in all cases by the end of the 90-day treatment period. The VAS for pain showed a 60% reduction after the first month and a 90% reduction by the end of the study.

Finally, no local or systemic adverse events related to the treatment were reported, highlighting the excellent safety profile of allogeneic cord blood serum eye drops (Table 2).

## 4. Discussion

This study provides strong evidence supporting the efficacy and safety of umbilical cord blood platelet lysate (UCB-PL) eye drops as a novel therapeutic option for managing severe ocular surface disorders associated with GvHD. GvHD is a significant complication of allogeneic HSCT, affecting a substantial proportion of patients and manifesting in both acute and chronic forms [4]. Chronic GvHD, in particular, is a leading cause of long-term morbidity and mortality among HSCT survivors, with ocular involvement being one of the most challenging aspects due to the complexity of symptoms and the impact on patients’ quality of life [9,27].

The ocular manifestations of GvHD, especially chronic GvHD, present a distinct therapeutic challenge [42]. This condition commonly affects the ocular surface, leading to severe DED, blepharo-kerato-epitheliopathy, and corneal ulcers [44]. These disorders are primarily driven by immune-mediated mechanisms that result in significant tissue damage and inflammation [40]. Conventional treatments, often based on immunosuppressive therapies such as corticosteroids, can be inadequate and come with substantial side effects that further compromise patients’ health and well-being [28,32]. In this context, the introduction of UCB-PL represents a promising advancement in the treatment landscape.

Our study’s findings indicate that UCB-PL eye drops offer significant therapeutic benefits, as evidenced by improvements in both patient-reported outcomes and clinical parameters. The substantial reduction in symptoms, as measured by the Ocular Surface Disease Index (OSDI), Symptom Assessment in Dry Eye (SANDE) questionnaire, and Visual Analogue Scale (VAS) score, underscores the potential of UCB-PL to improve patients’ quality of life. These improvements are not merely superficial but are supported by objective clinical assessments, including enhanced tear production (Schirmer Test), stabilization of the tear film (Break-Up Time, BUT), and an improvement in visual acuity (Best Corrected Visual Acuity, BCVA).

Furthermore, this study highlights the marked reduction in corneal ulcer size in patients with severe ocular GvHD. The fact that 88.8% of patients in Group III experienced a more than 50% reduction in ulcer size, with 77.8% achieving complete healing, is particularly compelling. This is significant considering the typically refractory nature of corneal ulcers in GvHD, which can lead to severe complications, including persistent epithelial defects (PEDs), scarring, and, ultimately, vision loss. The ability of UCB-PL to promote ulcer healing suggests a multifaceted mechanism of action, likely involving the modulation of the inflammatory environment and the promotion of epithelial and stromal repair.

The underlying mechanisms by which UCB-PL exerts its effects appear to involve the anti-inflammatory and regenerative properties of platelet lysate [11]. Platelets are rich in the growth factors and cytokines essential for wound healing and tissue regeneration. In the context of ocular GvHD, where inflammation and fibrosis are prominent features, UCB-PL may mitigate these pathological processes by reducing immune cell infiltration, modulating cytokine production, and enhancing reparative processes within the ocular surface [40,47]. This is supported by the observed reduction in clinical signs of inflammation, such as conjunctival redness and corneal neovascularization [52].

The safety profile of UCB-PL is another critical aspect of this study. GvHD patients are often subjected to long-term immunosuppressive therapy, which carries risks of significant side effects, including increased susceptibility to infections, organ toxicity, and secondary malignancies [32,62]. The fact that no local or systemic adverse events were reported in this study is highly encouraging, suggesting that UCB-PL could be a safer alternative or adjunct to existing therapies. This could be particularly beneficial for patients with severe ocular GvHD, for whom balancing effective immunosuppression and preserving overall health is especially delicate [33].

Despite the promising results, this study’s limitations must be acknowledged. The non-comparative design and relatively small sample size limit the generalizability of the findings. Additionally, the study was conducted in a single center, which may have introduced bias related to specific patient populations or clinical practices. Future research should aim to address these limitations by conducting larger, randomized, controlled trials across multiple centers. Such studies would provide more definitive evidence of the efficacy and safety of UCB-PL, allowing for the development of standardized treatment protocols.

Moreover, while the short-term benefits of UCB-PL have been demonstrated, the long-term effects of this therapy remain to be explored. It is essential to determine whether UCB-PL can sustain its therapeutic benefits over time and reduce the recurrence of ocular surface complications in GvHD patients. Long-term follow-up studies would also help assess the potential of UCB-PL to modify the course of the disease, potentially leading to better overall outcomes for patients with chronic GvHD [35].

Our study’s findings align with evidence from other international studies that have explored regenerative therapies for ocular complications of GvHD. In particular, our results are consistent with those of a recent international study by Pezzotta S. et al., in which they evaluated the long-term safety and efficacy of autologous platelet lysate drops in patients with ocular GvHD dry eye syndrome (DES) refractory to conventional therapies. Thirty-one patients were treated, with 51% completing 36 months of follow-up. At six months, all patients were classified as responders, showing significant improvements in their Glaucoma Symptom Scale (GSS) scores and Tear Break-Up Time (from 3 to 6 s), with results maintained over time. No severe adverse events occurred, supporting this treatment as a safe and effective long-term treatment option for oGvHD [63]. Another comparable study by Azari AA et al. documented promising results by retrospectively evaluating the effectiveness of autologous serum eye drops in treating dry eyes in 35 patients with ocular GvHD following stem cell transplantation. Their results showed that 55% of patients experienced symptom improvement, while 45% reported stability, with no significant adverse effects except one case of fungal keratitis. Corneal staining improved significantly (*p* = 0.003), and this approach was found to be safe and effective for managing dry eye symptoms in GvHD patients [64].

In general, the treatment of DED using allogeneic umbilical cord plasma eyedrops has shown promise in various studies. For instance, a recent study involving 40 patients showed, similarly to our study, significant improvements in kerato-epitheliopathy staining scores, the TBUT, and symptom relief (SPEED score). The results suggest that umbilical cord plasma could be an effective option for patients with treatment-resistant DED [65].

In 2012, a study had already highlighted the therapeutic potential of allogeneic serum eye drops for treating dry eye in patients with chronic graft-versus-host disease (cGVHD). This research demonstrated significant improvements in symptoms, tear osmolarity, corneal staining, impression cytology grade, goblet cell density, and tear breakup time (tBUT) after 4 weeks of treatment. Allogeneic serum was shown to be a safe and effective alternative when autologous serum is unavailable, with no notable side effects observed during follow-up [66].

This study provides compelling evidence that umbilical cord blood platelet lysate eye drops are a promising therapeutic option for patients suffering from severe ocular surface disorders due to GvHD. The positive outcomes observed, including significant symptom relief, improvements in clinical parameters, and the absence of adverse effects, highlight the potential of UCB-PL to become a standard treatment modality in this challenging patient population. However, further research is needed to confirm these findings and optimize treatment protocols to ensure the best possible outcomes for patients. If these results are confirmed in larger studies, UCB-PL could represent a significant advancement in managing ocular GvHD, offering new hope for improved patient care and quality of life.

## 5. Conclusions

In summary, this study presents strong evidence supporting the use of allogeneic UCB-PL eye drops as an effective and safe treatment for severe ocular surface disorders linked to GvHD. The data show notable improvements in both subjective symptoms and objective clinical parameters, suggesting that UCB-PL eye drops may offer a valuable therapeutic option for managing ocular GvHD. Given the current limitations of existing treatments and the encouraging results from this pilot study, further research is warranted to confirm these findings and to explore the broader application of UCB-PL therapy in other manifestations of GvHD, including acute ocular, mucosal, and cutaneous forms. This strategy holds potential for significantly enhancing the quality of life and clinical outcomes for patients affected by this challenging condition.

## Figures and Tables

**Table 1:** Patient groups.

Group	Number of Patients	Diagnosis
I	5	Severe Dry Eye Disease and filamentary keratitis
II	8	Severe blepharo-kerato-epitheliopathy
III	9	Corneal ulcers

**Table 2:** Clinical study parameters.

Parameter	Description
Mean Reduction in Ulcer Surface Area	Significantly greater reduction compared to baseline values at all assessment points
Final Assessment (Day 90)	88.8% of patients in Group III exhibited a reduction in ulcer size greater than 50%
Adverse Events	None reported

## Data Availability

The original contributions presented in the study are included in the article, further inquiries can be directed to the corresponding author.
